# Synchrotron X‐ray Radiography and Tomography of Vanadium Redox Flow Batteries—Cell Design, Electrolyte Flow Geometry, and Gas Bubble Formation

**DOI:** 10.1002/cssc.202000541

**Published:** 2020-06-02

**Authors:** László Eifert, Nico Bevilacqua, Kerstin Köble, Kieran Fahy, Liusheng Xiao, Min Li, Kangjun Duan, Aimy Bazylak, Pang‐Chieh Sui, Roswitha Zeis

**Affiliations:** ^1^ Karlsruhe Institute of Technology Helmholtz Institute Ulm Helmholtzstraße 11 89081 Ulm Germany; ^2^ Thermofluids for Energy and Advanced Materials (TEAM) Laboratory Department of Mechanical & Industrial Engineering University of Toronto Institute for Sustainable Energy Faculty of Applied Science & Engineering University of Toronto 5 King's College Road Toronto Ontario M5S 3G8 Canada; ^3^ School of Automotive Engineering Wuhan University of Technology Wuhan 430070 P.R. China; ^4^ Karlsruhe Institute of Technology Institute of Physical Chemistry Fritz-Haber-Weg 2 76131 Karlsruhe Germany

**Keywords:** carbon electrodes, electrolyte distribution, flow geometries, synchrotron X-ray imaging, vanadium redox flow cell

## Abstract

The wetting behavior and affinity to side reactions of carbon‐based electrodes in vanadium redox flow batteries (VRFBs) are highly dependent on the physical and chemical surface structures of the material, as well as on the cell design itself. To investigate these properties, a new cell design was proposed to facilitate synchrotron X‐ray imaging. Three different flow geometries were studied to understand the impact on the flow dynamics, and the formation of hydrogen bubbles. By electrolyte injection experiments, it was shown that the maximum saturation of carbon felt was achieved by a flat flow field after the first injection and by a serpentine flow field after continuous flow. Furthermore, the average saturation of the carbon felt was correlated to the cyclic voltammetry current response, and the hydrogen gas evolution was visualized in 3D by X‐ray tomography. The capabilities of this cell design and experiments were outlined, which are essential for the evaluation and optimization of cell components of VRFBs.

## Graphical abstract

**Now you see (through) me**: A modular vanadium redox flow cell is used to examine electrolyte distributions in carbon electrodes by synchrotron X‐ray imaging. The impact of three different flow geometries on the flow dynamics is studied, and electrochemical characterizations are concurrently performed with X‐ray imaging to visualize the hydrogen evolution. The unique capabilities of this cell design and experiments are outlined.

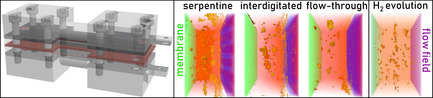

## Introduction

Solar, wind, and hydropower plants as so‐called green energy sources are naturally subject to fluctuating power output, resulting in the demand for energy conversion and storage systems. Redox flow batteries (RFBs) are capable of meeting these demands owing to the uncoupling of power and energy capacities, potentially low costs, fast response times, and a tunable design.^1^, ^2^, ^3^, ^4^, ^5^ The first vanadium redox flow battery (VRFB) was developed in the late 1980s and represents one of the most‐studied types of redox flow batteries owing to the low environmental impact of their materials, long cycle stabilities, and mostly harmless electrolyte cross‐contamination.^6^ The setup of a VRFB exhibits similarities to a polymer electrolyte membrane fuel cell, such as the ion‐exchange membrane, porous carbon electrodes, and flow field plates. However, the VRFB utilizes vanadium‐based electrolytes instead of reactant gases. When the battery is fully charged, the oxidation states of the vanadium ions are (II) and (V) in the anolyte and catholyte, respectively. Consequently, in the state of complete discharge the oxidation states are (III) and (IV). The corresponding redox reactions of the two half‐cells are shown in Equations (1), (2), together with the full‐cell in Equation (3), as well as the respective redox potentials.^1^, ^7^

(1) $$\mathrm{Anodic}\;\mathrm{half}\text{-}\mathrm{cell}:\;V{}^{3+}+e{}^{-}\vec{\leftarrow }V{}^{2+}E{}^{0}=-0.255\;V\;\mathrm{vs}.\;\mathrm{standard}\;\mathrm{hydrogen}\;\mathrm{electrode}\;\left(\mathrm{SHE}\right)$$

(2) $$\mathrm{Cathodic}\;\mathrm{half}\text{-}\mathrm{cell}:\;\mathrm{VO}{}^{2+}+H{}_{2}O\vec{\leftarrow }\mathrm{VO}{}_{2}{}^{+}+2\;H{}^{+}+e{}^{-}E{}^{0}=+1.004\;V\;\mathrm{vs}.\;\mathrm{SHE}$$

(3) $$\mathrm{Full}\text{-}\mathrm{cell}:\;\mathrm{VO}{}^{2+}+V{}^{3+}+H{}_{2}O\vec{\leftarrow }\mathrm{VO}{}_{2}{}^{+}+V{}^{2+}+2\;H{}^{+}E{}^{0}=+1.259\;V\;\mathrm{vs}.\;\mathrm{SHE}$$

The overall performance of the battery depends on several factors, such as the choice of the membrane,^8^, ^9^, ^10^, ^11^ the electrode material and pretreatment methods,^12^, ^13^, ^14^, ^15^ the electrolyte composition,^16^, ^17^ and the cell design, which includes the flow fields, the electrode structure,^18^, ^19^ and the degree of electrode compression.^20^, ^21^, ^22^, ^23^ Darling and Perry compared different flow field designs and discovered superior performance with interdigitated flow channels compared with a flow‐through configuration.^24^ Other sophisticated flow field designs have been presented in the literature to improve the overall system efficiency by reducing the pressure drop for a given electrode thickness. However, these designs would come at the expense of increased manufacturing complexity and system cost.^25^, ^26^, ^27^ Because the above‐mentioned redox reactions take place on the surface of the carbon felt electrodes, high wettability increases the accessible surface area and therefore facilitates the enhanced utilization of the electrode.^12^, ^28^, ^29^ Greco et al. examined the use of heat treatments to activate carbon paper electrodes and studied the resulting changes in properties.^29^ They report that although some properties, such as oxygen content and wettability, increase with increasing temperatures, the electrochemical activity decreases with heat treatment. As a result of this tradeoff, the peak overall cell performance was obtained with carbon felts treated at 475 °C.

Additionally, the hydrogen evolution at the anode as well as the carbon corrosion at the cathode are unwanted side reactions, which lead to performance losses because the evolved gases do not participate in further electrochemical reactions.^12^, ^30^, ^31^ Both reactions are shown in Equations (4), (5):

(4) $$\mathrm{Hydrogen}\;\mathrm{evolution}:\;2\;H{}^{+}+2\;e{}^{-}\to H{}_{2}↑E{}^{0}\leq 0.000\;V\;\mathrm{vs}.\;\mathrm{SHE}$$

(5) $$\mathrm{Carbon}\;\mathrm{corrosion}:\;C+2\;H{}_{2}O\to \mathrm{CO}{}_{2}↑+4\;H{}^{+}+4\;e{}^{-}E{}^{0}\geq 0.207\;V\;\mathrm{vs}.\;\mathrm{SHE}$$

The visualization of both the wetting behavior and the hydrogen gas evolution can lead to an improved understanding of the transport phenomena and thus provide important insights to further improve the VRFB cell design. Particularly suitable techniques for visualization include X‐ray or neutron radiography and tomography owing to the penetrating capabilities of the respective beams, which elucidate most of the common redox flow cell materials and designs. In earlier studies, Jervis et al. introduced a cell design to study the influence of compression on carbon felt materials by the means of an adjustable piston.^32^ This enabled the visualization of the carbon fibers, the vanadium electrolyte, as well as remaining air in three dimensions. In a follow‐up study with the same setup, they reported a non‐linear compression effect, in which the carbon felt material shows a higher porosity adjacent to the compressing piston in the center of the felt and a lower porosity towards the perimeter.^33^ Tariq et al. addressed the infiltration of vanadium solutions of several concentrations in previously dry carbon paper electrodes by utilizing time‐resolved 3D X‐ray tomography.^34^ They observed a non‐uniform infiltration of the electrolyte front owing to a strong anisotropy of the carbon fibers, and they reported the fastest infiltration was achieved with a VOSO_4_ electrolyte concentration of 0.5 m. Apart from X‐ray based techniques, Clement studied the local visualization of gas evolution and mass transport limitations of several carbon materials in VRFBs by employing neutron radiography.^35^ Our preceding study discussed the wetting behavior of the four vanadium species, as well as different compression ratios, and we showed the highest saturation values for V^III^ electrolytes and a decreasing trend in saturation for compressions above 50 %.^22^

We herein present a novel vanadium redox flow cell design and the experimental procedures to further investigate the impact of carbon felt wetting behavior and flow field design on the performance and hydrogen evolution behavior of the VRFB under potential control. This cell was specifically developed to utilize the visualization techniques at X‐ray synchrotron facilities, such as radiography and tomography. Compared with previous approaches,^14^, ^32^ this setup offers great flexibility in designing experiments for RFBs because it allows testing various types of electrode materials with different compression ratios and flow geometries (exchangeable flow fields) under potential control (in half‐cell and full‐cell configuration). Furthermore, the field of view is large enough to obtain technical relevant values and small enough to capture specific details such as gas bubbles growth.

## Results and Discussion

### Cell setup

**Figure 1:**
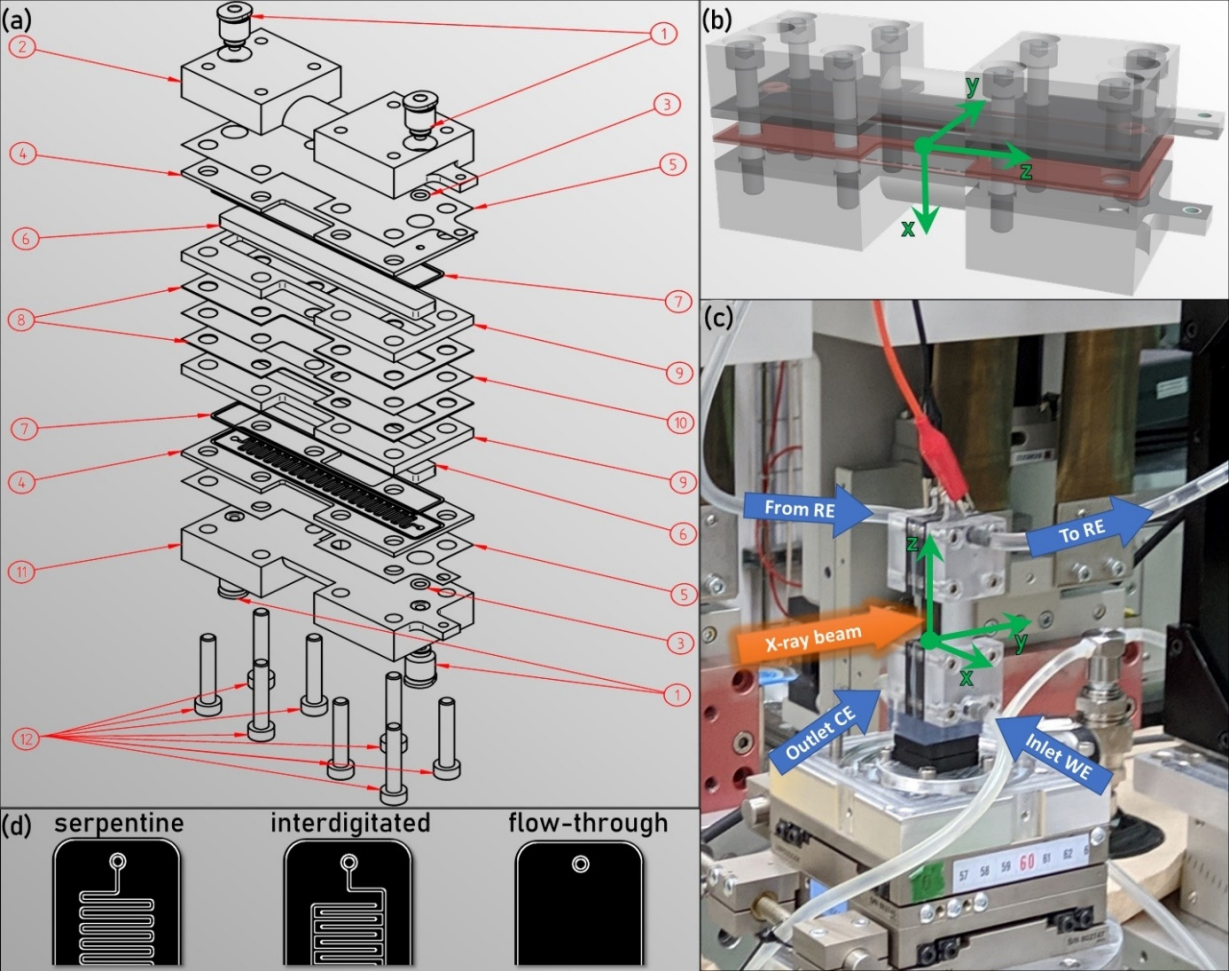
(a) Exploded view of the synchrotron cell. (1) Tube connectors, (2) endplate, (3) O‐ring gasket, (4) flow field, (5) stainless‐steel current collector, (6) carbon felt electrode, (7, 8) gaskets, (9) flow frame, (10) membrane, (11) endplate, (12) M4 screws. (b) 3D rendering of the assembled cell. (c) Photograph of the assembled and connected cell on the sample holder at the Canadian Light Source. (b, c) Including a coordinate system. (d) Representation of the top of the different flow field types.

We performed X‐ray radiography and tomography experiments based on a novel beamline half‐cell measurement setup, specifically designed for visualizing the electrolyte flow in VRFB electrodes. The design was adopted from a cell previously used to image fuel cells.^36^, ^37^, ^38^ To fit our needs, we implemented several modifications to enable the mounting of thicker electrodes, such as carbon felts with a thickness of up to 6 mm, modified the gasket design, and chose adequate materials for the flow frames, endplates, and tube connectors to ensure the tightness and stability towards the acidic electrolyte and avoid radiation damage of the materials to be examined. Figure 1 (a) shows an exploded view of the synchrotron cell with all components. Because all components are exchangeable, different types of flow fields can be used, that is, serpentine and interdigitated flow fields as flow‐by configurations, as well as a flat flow field as flow‐through configuration. Flow‐by configuration means that the electrolyte can flow by the electrode and through channels in the serpentine and interdigitated flow field structure, whereas the flow‐through configuration forces the electrolyte to travel through the porous electrode because there are no channels in the flat flow field. The channels of the serpentine and interdigitated flow fields are 0.8 mm wide and 0.8 mm deep and have a spacing of 0.8 mm. A representation of the different types of flow fields is shown in Figure 1 (d). The serpentine flow fields have an uninterrupted connection of the inlet and the outlet through the flow channels, which follow a zigzag path through the whole cell. In contrast, the flow channels of interdigitated flow fields do not directly connect the electrolyte inlet and outlet and thus force the electrolyte out of the channels and through the carbon felt. In the flow‐through configuration, no flow channels are available, and the electrolyte is forced to permeate the carbon felt electrode to reach the outlet. All flow fields are produced from graphite to ensure electrical conductivity and (electro‐)chemical stability.

Furthermore, exchangeable flow frames with different thicknesses define the compression ratio of the carbon felt and can be adjusted as needed. The flow frames accommodate carbon felt electrodes with dimensions of up to 80 mm in height and 8 mm in depth, which are defined throughout this study as the *z*‐direction and *y*‐direction, respectively. The latter constitutes the thickness of the electrode in the traveling direction of the X‐ray beam. The *x*‐direction is defined by the width of the field of view, which is 15.8 mm wide and captures the whole cell including the flow fields, even when housing an electrode with the maximum thickness of 6 mm on each side. The height of the electrode (80 mm) is significantly larger than the height of the field of view (8.5 mm). This was chosen deliberately to exclude edge effects and to capture solely the distribution and flow behavior in the bulk electrode. Hence, we obtain a region of interest of our electrode with the dimensions (8.5×8×*d*) mm^3^, in which *d* is the thickness of the electrode. This covers the representative elementary volume, as was shown before by Fishman et al.^39^ and George et al.^40^ Because edge effects also occur along the membrane and the flow field, 0.75 mm of the electrode was omitted from the calculation of the bulk saturation values. In this study, the saturation is defined as the volume fraction of the region of interest (*V*_ROI_), which is flooded by the electrolyte (*V*_electrolyte_), as shown in Equation (6):

(6) $$Saturation=\frac{V_{electrolyte}}{V_{ROI}}\ln\left(\frac{I_{0}}{I}\right)$$

To control the applied potential and current at one of the carbon felt electrodes, we utilize a half‐cell setup: herein, the electrolyte is injected through one electrode of the cell from the bottom to the top by using a syringe pump (working electrode), followed by tubing from the outlet of the first electrode to a reference electrode (Ag/AgCl reference electrode), and then directly connected to the top of the other electrode (counter electrode), as indicated by the arrows in the cell setup shown in Figure 1 (c). This setup is similar to the double half‐cell (DHC), which was previously reported in the literature.^24^, ^41^, ^42^ Additionally, Figure 1 (b, c) includes a coordinate system colored in green, which marks specific directions inside the working electrode and the cell. The origin of the coordinate system is positioned inside the cell at the membrane at the bottom of the field of view. From there, the *x*‐axis points towards the flow field of the working electrode through the compressed carbon felt, whereas the *y*‐axis represents the depth of the carbon felt, which is penetrated by the X‐ray beam. The *z*‐axis indicates the height of the cell along the membrane and is limited by the field of view (8.5 mm). Thus, we obtain images with *x*,*z* coordinates in radiography experiments, whereas the tomograms are depicted in the *x*,*y* and *y*,*z* directions.

### Injection experiment

To investigate the influence of our flow field configurations on electrolyte saturation, vanadium(IV) electrolyte was injected into dry carbon felt electrodes with a steady flow rate of 500 μL min^−1^ for 300 s (injection). After a subsequent 300 s relaxation period, a tomogram was recorded. This was followed by an additional electrolyte injection into the sample at a higher flow rate of 30 mL min^−1^ for 60 s (flow) to simulate the operation of a full‐cell. This step ensures that the cell is in a state resembling the steady‐state operation of an operating redox flow system. The sequences during injection and flow were recorded by radiography at 25 frames per second (fps). The investigated flow fields include a serpentine, an interdigitated, and a flat flow field.

**Figure 2:**
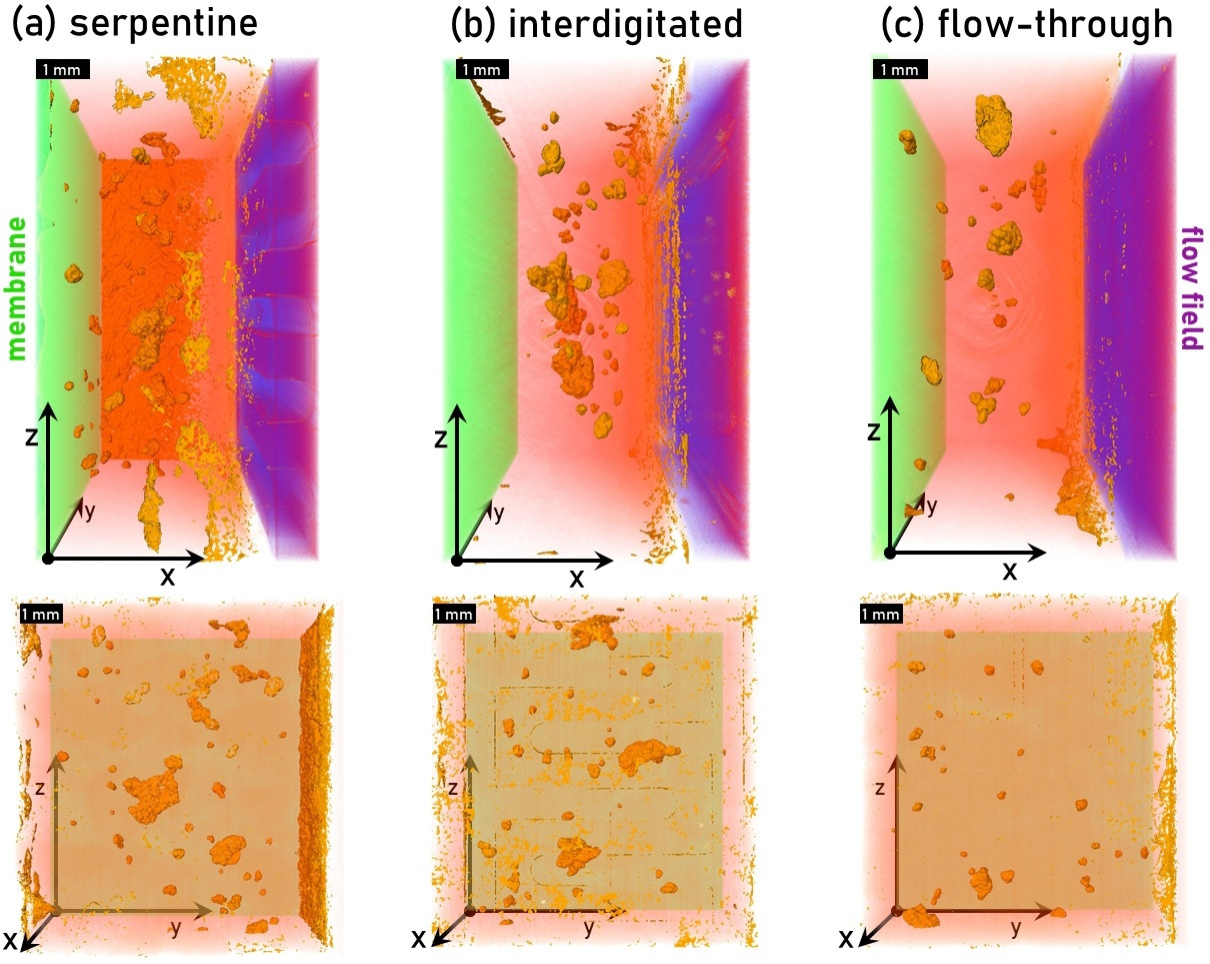
Visualization of the remaining air in the carbon felts by the means of X‐ray tomography after the injection of the vanadium electrolyte through (a) serpentine and (b) interdigitated flow fields, as well as (c) a flow‐through configuration. The membrane is depicted on the left (green), the flow field on the right (purple), and the remaining air pockets in between are displayed in orange. The origin of the coordinate is at the same position in the top and bottom row.

Figure 2 displays the remaining air in the carbon felts for each flow field configuration visualized by the means of tomograms recorded after the injection at 500 μL min^−1^ for 300 s. The images show the 3D space of the porous electrode from the membrane (left, green) to the flow field (right, purple) throughout the whole field of view. The trapped air inside the electrode is colored in orange, and the shades of orange signify the *y*‐position of the air bubble, in which the darker shades correspond to higher *z*‐coordinates. The accumulation of gas bubbles along the flow field is visible for each flow field, even though the effect is reduced in the case of the flat flow field. By using flat flow fields, the electrolyte is forced through the porous electrode and cannot flow through the flow field channels, as is the case with interdigitated or serpentine flow fields, for which, in the latter configuration, the inlet and outlet of the cell are even connected directly by the flow field channel [see Figure 1 (d)]. This increases the saturation of the electrode not only in the vicinity of the flow field but also in the electrode itself.

**Figure 3:**
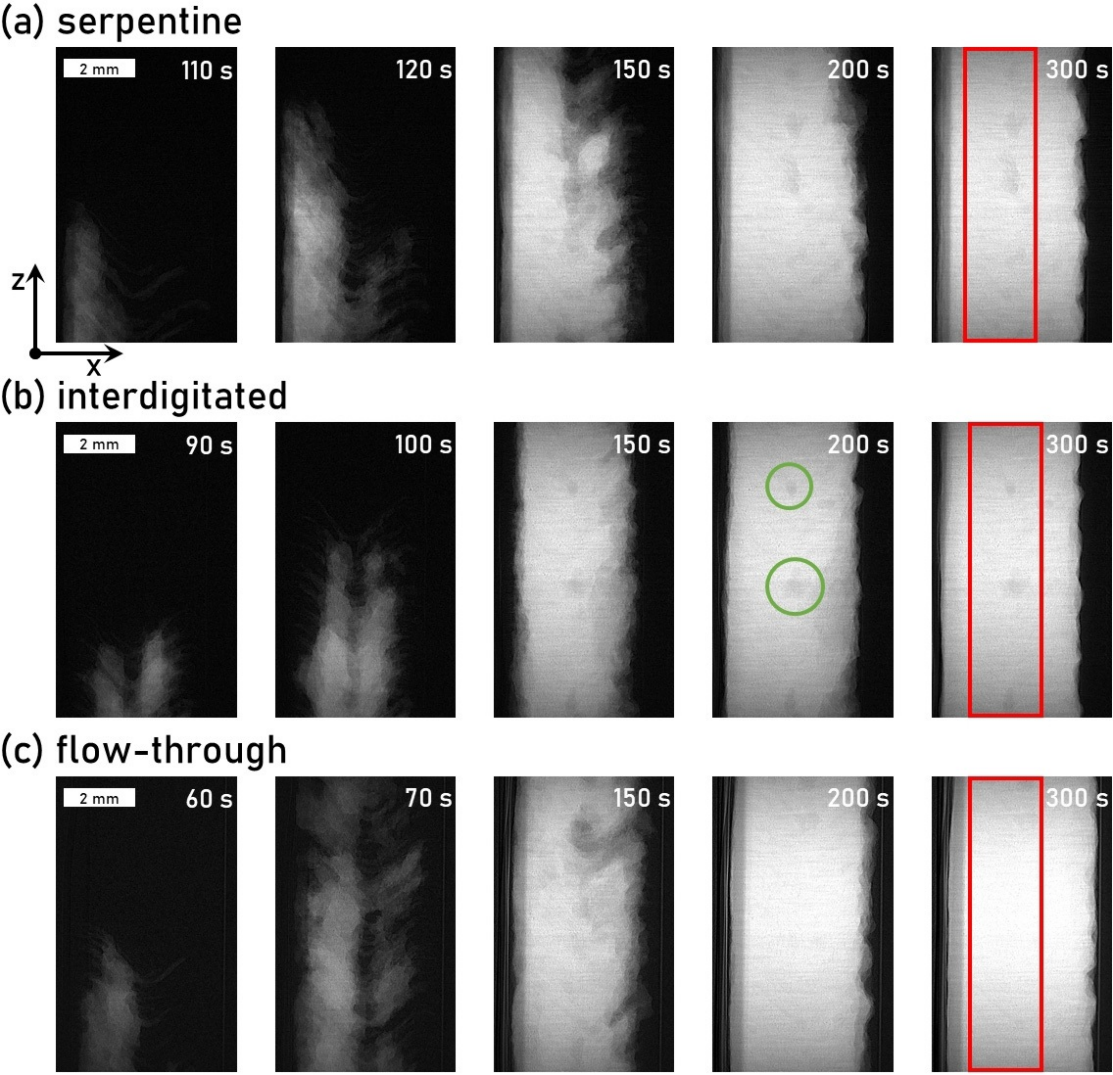
Visualization of the vanadium electrolyte at specific times during its injection by the means of X‐ray radiography through (a) serpentine and (b) interdigitated flow fields, as well as (c) a flow‐through configuration. The brighter the image is, the higher the electrolyte thickness at this point. Darker spots signify the presence of trapped air bubbles inside the porous electrodes; two examples are marked with a green circle in (b). The red box in each last image shows the region of interest, which was selected to calculate the average saturation.

The radiograms in Figure 3 show the difference between the image at a given time of the injection and the image of the dry cell and display changes in the electrolyte distribution for the different flow fields during the injection. The first image (left) of each flow field shows the radiograms at the point shortly after the electrolyte enters the field of view and the electrolyte front reaches one third along the *z*‐direction of the field of view. The second radiogram shows the injection 10 s after the first radiogram, followed by three radiograms at 150, 200, and 300 s after the start of the injection. The local brightness (highest grayscale value) of the image corresponds to the electrolyte thickness at this point. The brighter the image, the more electrolyte was traversed by the X‐ray beam. Dark round spots signify the presence of trapped air bubbles inside the porous electrodes.

Hereby, we can observe the difference of the electrolyte invasion speed: the electrolyte front is already visible after 60 s with the flow‐through configuration (a), followed by interdigitated (b) and serpentine (c) flow fields (90 and 110 s). 10 s later, the electrolyte front has already reached the top of the field of view in (c), whereas, in (a) and (b), it has only reached approximately 60 % of the height in the field of view. Interestingly, the electrolyte invasion mainly proceeds in the center of the felt or even closer to the membrane on the left side of each radiogram. This may be a result of the structure of the specific carbon felt used in our experiments (GFA6EA), which shows a pronounced seam or edge in the center of the felt resulting from the manufacturing. This is even clearer in the second radiograms, especially in (b) and (c), in which a region in the center of the felt is not invaded by the electrolyte. After 150 and 200 s, the felt is already saturated to a large extent, although (a) still shows a large unsaturated area in the top right corner of the field of view. In each configuration, after 300 s, the whole carbon felt is visibly saturated.

To quantify the average saturation, a region of interest in the bulk of the electrode was selected (red rectangle in the right images of Figure 3) and processed as described in the Experimental Section. We observed the highest saturation for flat flow fields (96.56 % after 500 μL min^−1^, 99.42 % after 30 mL min^−1^), which is also visible in the tomograms in Figure 2 because the number of trapped air bubbles in the center of the electrode is smaller than the other flow field configurations. The cells with serpentine and interdigitated flow fields showed a similar saturation after the initial injection at 500 μL min^−1^ (90.29 and 90.99 %). However, after the increased flow with 30 mL min^−1^, the saturation of the serpentine flow field increased more strongly than in the case of the interdigitated flow field (99.27 vs. 92.96 %). The saturation values after injection and flow are summarized in Table 1.

**Table 1:** Saturation values for each cell configuration after the injection and flow.

Flow field configuration	Saturation after injection at 500 μL min^−1^ [%]	Saturation after flow at 30 mL min^−1^ [%]
serpentine flow field	90.29	99.27
interdigitated flow field	90.99	92.96
flow‐through	96.56	99.42

### Cyclic voltammetry

The visualization of the electrolyte‐filled carbon felt electrode during a potential sweep induced redox reaction can provide novel insights into optimization approaches. To provide an example of this experimental procedure, this chapter only focuses on the cyclic voltammograms of the flow‐through setup (flat flow field).

**Figure 4:**
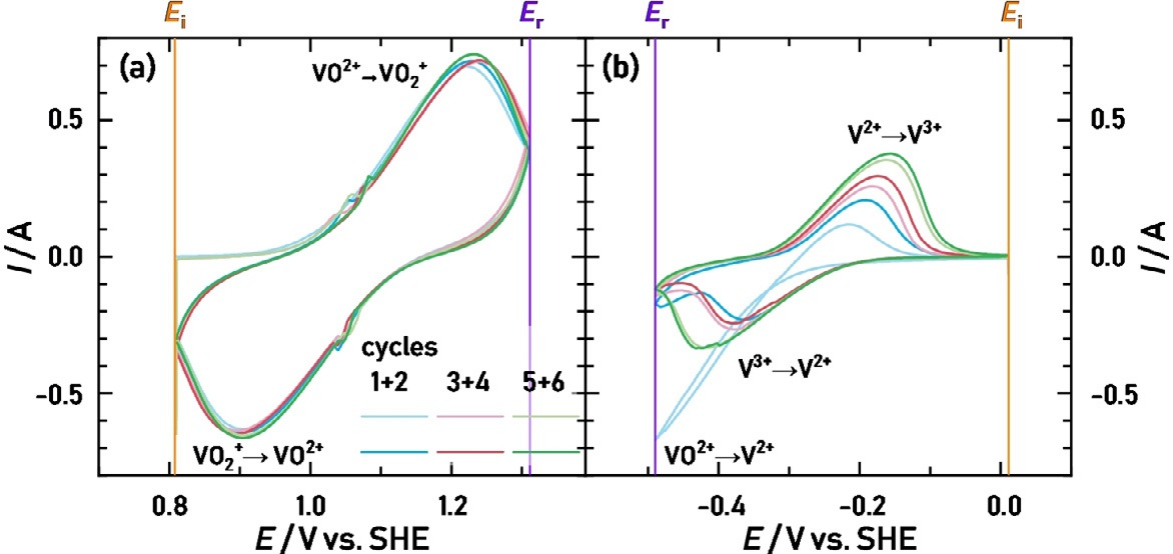
Cyclic voltammograms of the carbon felt in the vanadium(IV) electrolyte in the potential region of (a) approximately 0.8 to 1.3 V vs. SHE and (b) approximately −0.5 to 0.0 V vs. SHE. Two cycles were recorded, followed by a potential hold during a tomography recording at OCV. This procedure was repeated three times. The scan rate was 10 mV s^−1^. The initial (*E*_i_, orange) and reverse potentials (*E*_r_, violet) are highlighted.

In Figure 4, we show the cyclic voltammograms of (a) the VO^2+^/VO_2_^+^ and (b) the V^2+^/V^3+^ redox couple and include reference lines at the initial cycling potential (*E*_i_) and the reverse potential (*E*_r_). For (a), pronounced oxidation and reduction peaks appear immediately in the first cycle and show a similar magnitude of peak currents (0.740 A for the oxidation peak, 0.662 A for the reduction peak), which points towards good reversibility of the redox reaction. After two cycles, we set the potentiostat to the open‐circuit voltage (OCV) while acquiring the tomogram. This procedure was repeated three times. Although the cyclic voltammetry measurement was interrupted twice during this experiment, a stable redox peak was obtained because the electrolyte flow was stopped, and no leakage of the cell was observed. This is even more clear in the V^2+^/V^3+^ redox measurement in Figure 4 (b), because the vanadium(IV) electrolyte was reduced to vanadium(II) during the cathodic scan of the first cycle, and therefore two electrons were transferred, resulting in negative currents twice as large in magnitude as the subsequent cycles with pronounced V^2+^/V^3+^ redox peaks, which was previously described.^30^, ^31^, ^43^ Again, the measurement was interrupted by an OCV period after two cycles, but unwanted oxidation of vanadium(II) or (III) was not observed because the redox peak currents were increasing in magnitude with each cycle. This increase of the peak current densities can be ascribed to the increasing amount of V^2+^, which then is oxidized to V^3+^. With each cycle, more VO^2+^ electrolyte gets reduced to V^2+^ inside the confined space of the cell, resulting in increasing peak currents. Similar oxidation and reduction peak currents were also observed for this redox couple, indicating good reversibility of the redox reaction.

**Figure 5:**
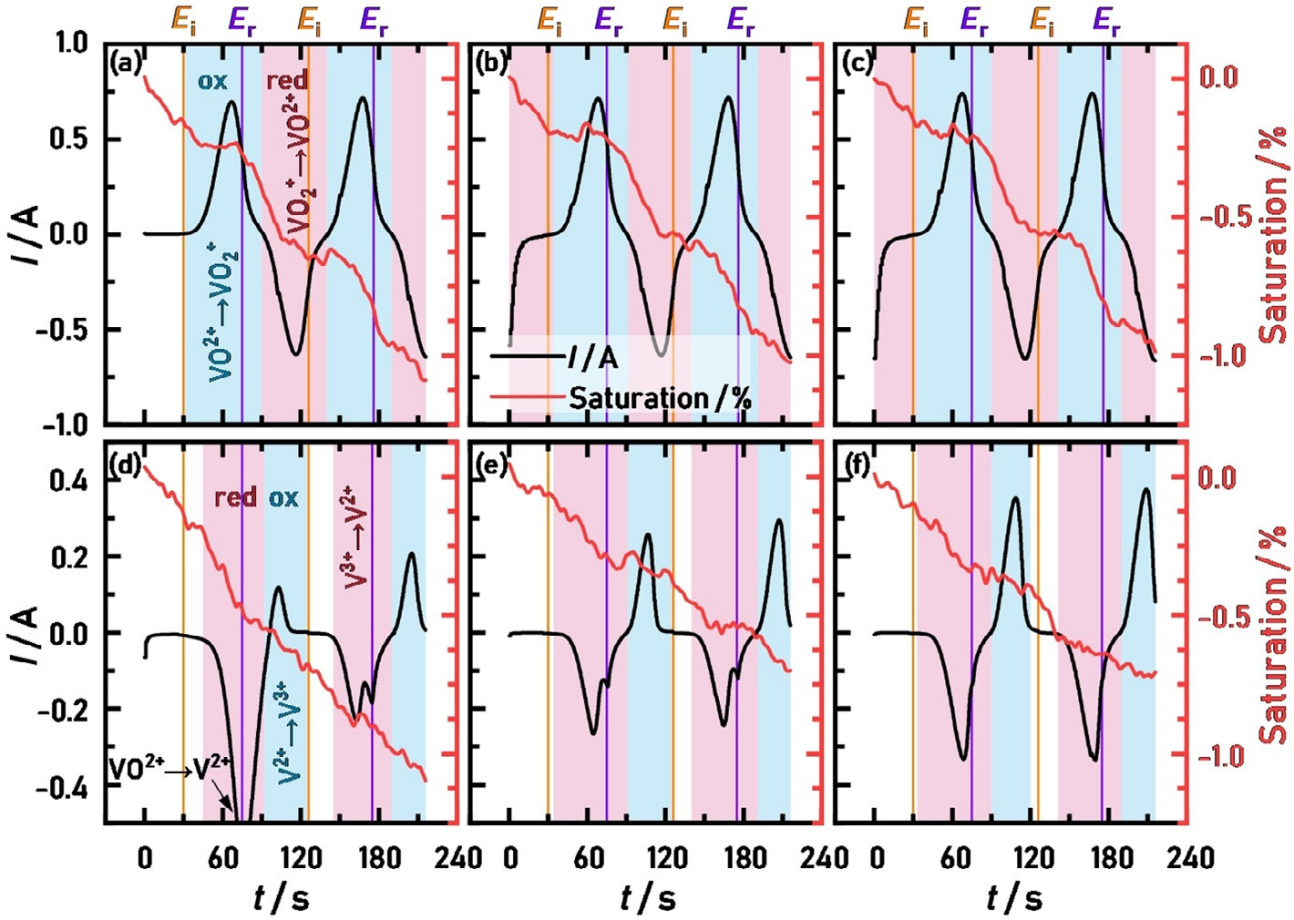
Changes in the average saturation (red line, with LOWESS applied) and the current response (black line) of the carbon felt during potential cycling between 0.8 and 1.3 V vs. SHE (top row, VO^2+^/VO_2_^+^ redox couple), and −0.5 and 0.0 V vs. SHE (bottom row, V^2+^/V^3+^ redox couple) for the respective cycles 1 and 2 (a, d), 3 and 4 (b, e), and 5 and 6 (c, f). Furthermore, the periods of oxidation (blue) and reduction (red) are highlighted. The scan rate was 10 mV s^−1^. The initial (*E*_i_, orange) and reverse potentials (*E*_r_, violet) are highlighted.

During the cyclic voltammetry measurements, time‐resolved radiograms were obtained at 25 fps. To minimize the influence of possible membrane movement and edge effects, we utilized a 2.8 mm×8.3 mm section within the working electrode as a representative area to calculate the average saturation for each frame. With this procedure, we could correlate the changes in saturation with the measured current during the cyclic voltammetry (see Figure 4) over time, as shown in Figure 5 (a–c) for the VO^2+^/VO_2_^+^ and (d–f) for the V^2+^/V^3+^ redox couples. To increase the visibility of the data, a LOWESS (locally weighted scatterplot smoothing) was applied to the average saturation values, which reduces the data noise without obscuring the trend of the data.

An overall decrease in the average saturation was observed during the cycling, with the strongest decrease of −1.09 % after the first two of the positive and negative cycles. The subsequent cycles showed a very similar saturation decrease in each case of approximately −1.00 (positive) and −0.70 % (negative). This decrease of the average saturation can be ascribed to the carbon corrosion [CO_2_ evolution, see Eq. (5)] during the positive cycles and hydrogen evolution [Eq. (4)] during the negative cycles because the applied potentials are well within the potential window of these side reactions, which are above 0.21 V (vs. SHE) for the carbon corrosion and below 0.0 V (vs. SHE) for the hydrogen evolution. As our previous studies showed by the means of differential electrochemical mass spectrometry (DEMS),^30^, ^31^ the strongest CO_2_ and H_2_ evolution occur during the first cycles of freshly prepared carbon felts, which could explain the stronger decrease of the average saturation during the first two cycles. Because the changes in the average saturation are relatively small, 3D representations of the carbon felt by the means of tomography do not provide additional information and are consequently not shown.

Additionally, we observed changes in the slope of the saturation during all cycles. During the positive cycles in Figure 5 (a–c), the slope was almost zero at each first oxidation period (highlighted in blue) and more negative during reduction periods (red), resulting in a step‐like shape.

During the first 30 s, the potential was held at *E*_i_, which resulted in a decrease in the average saturation. For *E*_i_=0.8 V in Figure 5 (a), a small positive current of approximately 1 mA could be measured, which originated from the already present carbon corrosion. As soon as the cycling began and higher potentials were applied, the change in the saturation stabilized because the VO^2+^ was now preferably oxidized. As the concentration of VO^2+^ decreased after reaching the reverse potential *E*_r_, the average saturation also showed a strong decrease. The same also applies to the reduction of VO_2_^+^. Besides the side reaction, the changing vanadium species also influences the saturation because VO_2_^+^ electrolytes showed a 5 % higher saturation than VO^2+^ owing to increased wetting properties, as we observed in our previous study by the means of injection experiments.^22^

The changes in the average saturation during negative cycles in Figure 5 (d–f) cannot be correlated to the applied potential or resulting current as easily because the step‐like shape is far less pronounced and the electrolyte composition changes with each cycle, as described above.

During the first 30 s in Figure 5 (d), for which a constant potential of *E*_i_=0.0 V was applied, the average saturation already decreased steadily, presumably owing to the reduction of surface functional groups, such as quinones, as well as a partial reduction of VO^2+^ to V^3+^, which both might influence the wetting properties.^22^, ^44^ During the first reduction period, in which VO^2+^ was reduced to V^2+^, the slope average saturation decreased owing to strong hydrogen evolution, which was additionally catalyzed by V^2+^, as observed in our previous DEMS studies^30^, ^31^ and investigated in detail by Lee et al.^43^ In the following cycles in Figure 5 (e, f), the peaks of the V^2+^/V^3+^ redox reaction became more pronounced, and the average saturation almost flattened at potentials around the reverse potential of *E*_r_=−0.5 V. Whereas in the first cycles of Figure 5 (e, f), this flattening occurred only during the positive scan, that is, after reaching *E*_r_, the average saturation remained unchanged during the reduction of V^3+^ (red periods) in the respective second cycles. As a side note, the first of the two negative peaks during the reduction periods (except for the very first reduction) in Figure 5 (d–f) results from the reduction reaction of V^III^ to V^II^, whereas the second peak results from the potential reverse at *E*_r_ of the cyclic voltammogram sweep, which interrupts the exponential decrease of the hydrogen evolution. However, a more detailed interpretation of the correlation between the saturation and the cyclic voltammetry of the V^2+^/V^3+^ redox couple is beyond the scope of this study and requires a detailed experimental procedure, such as the variation of the vanadium concentration or electrolyte composition.

In general, cyclic voltammetry measurements are feasible with this setup, and the simultaneous radiography provides additional insight into the physical and (electro‐)chemical behavior of the VRFB system. In future works, more advanced procedures could be applied, such as different scan rates to gain information about the transient response of the redox system, or long‐term cycling behavior to investigate the electrochemical stability of the carbon felt electrodes.

### Hydrogen evolution reaction

As discussed before, the hydrogen evolution reaction is one of the main disadvantageous side reactions of the VRFB. By applying a constant potential of −0.3 V (vs. SHE) for 300 s, we induced a continuous hydrogen evolution, again followed by the acquisition of a tomogram at steady‐state OCV to visualize hydrogen bubbles. This part only includes the flow‐through setup as an example, which utilizes the flat flow fields.

**Figure 6:**
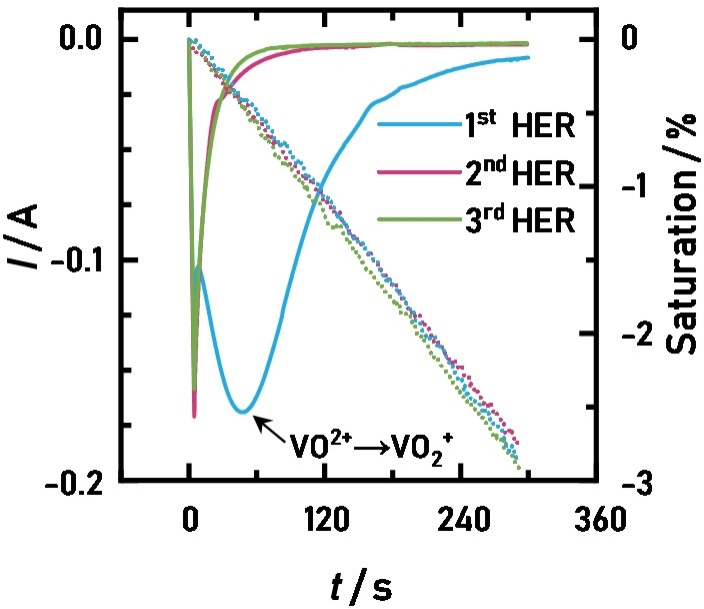
Time‐resolved current response of the three hydrogen evolution periods and the decrease of the average saturation (dotted line, with LOWESS applied) at a potential of −0.3 V vs. SHE. The peak during the first period corresponds to the reduction of VO^2+^ to V^2+^.

The resulting current response and the calculated average saturation for the three consecutive hydrogen evolution periods are shown in Figure 6. Again, the raw data of the average saturation was processed with LOWESS to improve the visualizing of the trends in the data.

During the first HER period, a pronounced negative current peakwa observed, which corresponds to the reduction of the vanadium(IV) electrolyte to vanadium(II), whereas the following two periods showed a very similar exponential decay of the current. The saturation showed a slightly less negative slope during the reduction of the vanadium, as was also visible in Figure 5 (d), which points towards the influence of the vanadium species on the wetting. After the reduction, the slope of the average saturation leveled off and reached a constant slope, similar to what was observed for the two consecutive HER periods. Overall, a final saturation decrease of almost 3 % was reached during all three hydrogen evolution periods.

**Figure 7:**
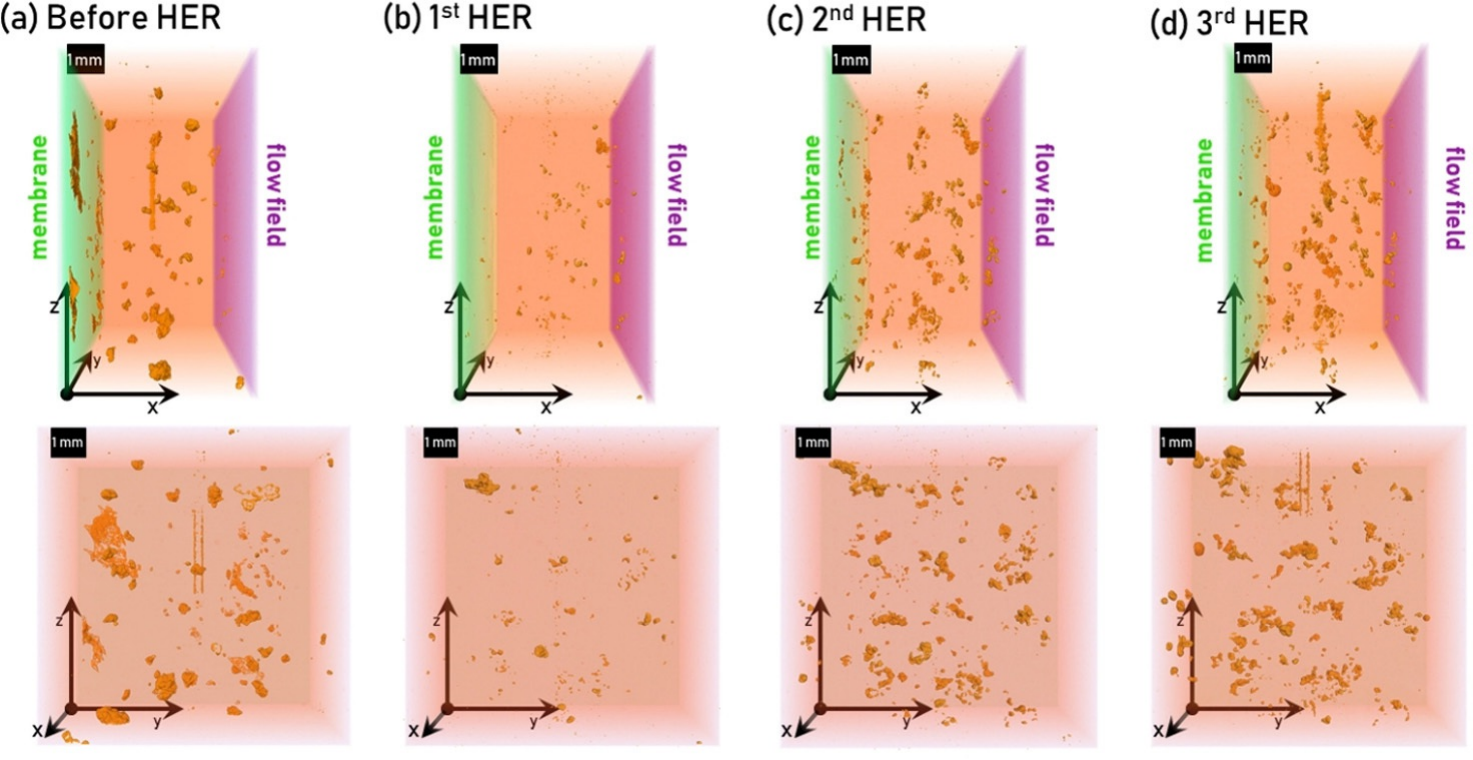
Tomograms showing (a) the remaining air before HER, and (b–d) the evolved gas after each hydrogen evolution period inside the carbon felt working electrode, viewed from the front of the cell (*x*,*z*, top row) and the membrane face (*y*,*z*, bottom row). In the top row, the membrane is displayed on the left (green), the flow field on the right (purple). The gas bubbles are shown in orange. The origin of the coordinate is at the same position in the top and bottom row.

Between each HER period, the potentiostat was set to OCV to provide stationary conditions, and tomograms of the cell were recorded to visualize the growth of the hydrogen formation. In Figure 7, the development of the hydrogen bubble formation is shown, viewed from the top of the cell in the top row and the membrane face in the bottom row. Some gas bubbles are already visible in Figure 7 (a), before the HER, and are assumed to be trapped air bubbles. The orange bubbles displayed in each subsequent image are the bubbles formed solely during the respective preceding HER period. This was achieved by subtracting the tomogram recorded before an HER period from the tomogram that was recorded after. As an example, the tomogram in Figure 7 (b) results from the difference between the tomograms before [Figure 7 (a)] and after the first HER (not shown), and thus only shows the bubbles that formed during this first HER period.

A growth of hydrogen bubbles in the center of the felt is visible, which is expected owing to the low solubility and high hydrophobicity of H_2_. With each HER period, more evolution sites appear, and the existing bubbles grow further, which is reflected in the increased amount of voxels (13 μm/voxel) in the tomogram and results in a bubble volume increase of 0.21, 0.48, and 0.65 mm^3^ during the first, second, and third HER periods, respectively. Because vanadium was reduced first during the first HER period, the hydrogen formation was slightly higher during the second HER (0.27 mm^3^) and lower again during the third HER period (17 mm^3^), which correlates with our previous DEMS study, in which the highest amount of hydrogen was formed after the vanadium reduction.^30^

The tracking and visualization of hydrogen evolution are possible with this setup, and unique insights into the processes of VRFBs are accessible. With further, more advanced experiment procedures, detailed studies can be performed, and even full‐cell charge/discharge experiments can be visualized.

## Conclusions

In this study, we introduced a vanadium redox flow battery (VRFB) cell design for synchrotron X‐ray radiography and tomography to visualize the injection behavior of the vanadium electrolyte through carbon felt materials. By the means of exchangeable flow‐by and flow‐through geometries and manifolds, we could investigate the impact on the flow dynamics. Additionally, we studied the influence of electrochemical measurements on the electrolyte saturation of the carbon felt by the means of cyclic voltammetry experiments and constant potential measurements to achieve a controlled hydrogen evolution.

After injecting a vanadium electrolyte with a steady flow rate into the carbon felts, we observed the highest saturation of the electrode with a flow‐through configuration (96.56 %), whereas serpentine and interdigitated flow fields led to similarly saturated carbon felts (90.29 and 90.99 %). An additional electrolyte flow with a higher flow rate increased the saturation to up to 97 % in the case of serpentine flow fields.

By the means of a modified DHC setup, we were able to record cyclic voltammograms for the VO^2+^/VO_2_^+^ and V^2+^/V^3+^ redox couples and simultaneously track the changes in the average saturation in the carbon felt working electrode. We observed an overall decrease in the average saturation of up to −1.09 % during the redox cycles owing to side reactions, such as carbon corrosion and hydrogen evolution. Furthermore, the changes in the slope of the time‐resolved average saturation could be correlated to the current response of the cyclic voltammetry and therefore to the polarization of the electrode surface, the vanadium species, and the intensity of the occurring side reactions.

The setup allowed us to investigate the presence of gas bubbles owing to parasitic side reactions. In a constant potential experiment, we tracked the hydrogen evolution reaction by its current response and the time‐resolved decrease in saturation and additionally visualized the gas evolution in 3D by tomography. We showed that the evolved hydrogen gas mainly accumulates at pre‐existing air bubbles, which underlines the importance of a high wettability of the electrode surface.

Hence, the cell design introduced in this work shows promising results to study the wettability during electrolyte injection and electrochemical measurements, as well as to visualize side reactions inside the carbon felt electrode. More advanced experimental procedures, such as full‐cell charge/discharge measurements or detailed compression studies are entirely feasible with this setup and may be included in future work. These measurements will help us develop theoretical models for a better understanding of the multiphase and interfacial flow phenomena within the porous electrode. Furthermore, these experiments are essential for the evaluation and optimization of cell components currently being used in VRFBs.

## Experimental Section

### Synchrotron setup

The radiograms and tomograms were obtained at the Biomedical Imaging and Therapy Bending Magnet (BMIT‐ID) 05ID‐2 beamline at the Canadian Light Source Inc. (Saskatoon, Saskatchewan, Canada).^45^ For the radiograms, an exposure time of 50 ms, a frame rate of 25 fps, and an effective pixel size of 13 μm were utilized. A Budker superconducting wiggler was used to generate high‐intensity monochromatic synchrotron radiation with an energy of 30 keV in the storage ring. The distance between the sample and the light source was 58 m, and the sample and the detector were separated by 40 cm. An indirect detection scheme was applied, in which the luminescent image produced by the X‐rays was captured in a 500 μm thick YAG scintillator coupled with a CMOS camera (Orca Flash V2 sCMOS, Hamamatsu Photonics, Shizuoka, Japan). The YAG scintillator was chosen owing to its fast decay time and its low afterglow. The resulting field of view was 26.6 mm horizontally and only 8 mm vertically, as the vertical field of view was limited by the divergence of the synchrotron beam. The estimated spatial resolution of the detector was 40 μm. Ten dark‐field images (camera background, no beam) and ten flat field images (background with beam, no sample) were recorded before and after each tomographic set of images, during which the sample was rotated 200° around its vertical axis. The 2000 images for the three‐dimensional tomograms were only recorded within 180° to minimize the effect of acceleration and deceleration during rotation, resulting in one image every 0.09°. The images were reconstructed based on the script *ufo‐kit*, developed at the Karlsruhe Institute of Technology.^46^, ^47^ To remove ring artifacts in the raw images, low pass filtrating sinograms in the frequency domain were applied before further reconstruction steps were performed.

### Determination of the attenuation coefficient

The X‐ray attenuation coefficient of the V^IV^ electrolyte at a beam energy of 30 keV was determined by performing a calibration based on the Beer–Lambert law. This experiment was designed after the example of Ge et al., who showed a schematic drawing of the calibration cell and calculated the attenuation coefficient of water at the same beamline as this work.^48^ The step‐wise increase of the electrolyte thickness and the constant material thickness of the device in the beam path allowed plotting ln (*I*_0_/*I*) versus the thickness of the electrolyte and yields a straight line, in which *I*_0_ is the empty calibration device and *I* is the local intensity of the filled calibration device. The slope of the regression amounted to the attenuation coefficient *μ* for the electrolyte at this specific energy. Image correction (dark field and flat field correction, and the beam intensity decay correction) was applied as described in the section “Experimental procedures and data processing”. The resulting attenuation coefficient amounted to *μ*=0.554 cm^−1^ with an *R* ^2^ of 0.9999.

### Carbon materials, vanadium electrolyte, instruments

The carbon felt materials in this study were supplied by SGL Carbon (Meitingen, Germany). We used Sigracell® GFA 6 EA, a Rayon‐based, graphitized carbon felt with a nominal thickness of 6 mm compressed to 4.5 mm (25 % compression ratio) in all experiments. The carbon felts were pretreated in a muffle oven for 25 h at 400 °C in an air atmosphere to introduce active sites on the carbon surface.^31^

The vanadium(IV) electrolyte was prepared by dissolving 0.1 m VOSO_4_ (VOSO_4_ **⋅**5 H_2_O, chemically pure, GfE) in 2 m H_2_SO_4_ [Suprapur®, Merck, diluted with purified Milli‐Q water (18.2 MΩ⋅cm)]. This electrolyte was bubbled with nitrogen to remove atmospheric air and subsequently transferred to a syringe before each experiment. The syringe was placed in a syringe pump (LA‐100 by Landgraf HLL, Germany), and the potential in the electrochemical measurements was controlled by the means of a Bio‐Logic SP‐300 potentiostat. We utilized a Nafion® N117 membrane and an Ag/AgCl reference electrode (3.5 m NaCl, *E*=0.209 V vs. SHE at 25 °C). All potentials in this study were converted to SHE.

### Experimental procedures and data processing

To study a wide range of properties of the carbon felt, as well as the capabilities of the cell itself, the following experimental procedure was performed.

**Injection**: In each cell configuration, we prepared a cell with completely dry electrodes and recorded a tomogram, followed by an injection of the electrolyte (500 μL min^−1^ for 300 s) from the bottom of the cell at the working electrode while performing radiography. After a relaxation period of 300 s, a tomogram of the wet cell was recorded subsequently.

**Flow**: After the injection a flow procedure was performed with 30 mL min^−1^ for 60 s with simultaneous radiography, followed by another relaxation period of 300 s. A tomogram of the fully flooded cell was recorded subsequently.

**Cyclic voltammetry**: Radiography was performed during a potential hold of 30 s at an initial potential (*E*_i_) of approximately 0.8 V vs. SHE, followed by two potential sweeps between *E*_i_ and the reverse potential (*E*_r_) of approximately 1.3 V vs. SHE for the VO^2+^/VO_2_^+^ redox couple. For the V^2+^/V^3+^ redox couple, the following potentials were utilized: *E*_i_≈0.0 V vs. SHE and *E*_r_≈−0.5 vs. SHE. After the two cycles, a tomogram was recorded while holding the cell at the OCV. This procedure was repeated three times for each potential region.

**Hydrogen evolution**: The hydrogen evolution was studied by applying a potential of approximately −0.3 V vs. SHE for 300 s and simultaneously recording radiograms, followed by an OCV period of 300 s and a recording of a tomogram. This procedure was repeated three times.

**Calculation of the average saturation values**: Radiograms were recorded at 25 fps and required post‐processing to reduce the background noise and the camera noise because they were the input for the Beer–Lambert law [Eq. (7)] to determine the local thickness of the electrolyte (*d*_el_) in the image, as previously reported by Ge et al.^49^ Here, *I*_0_ describes the initial intensity and *I* the current intensity of the detected beam, which corresponds to the grayscale values of the radiograms, whereas *μ* is the attenuation coefficient and *ϕ*_r_ is the porosity given by the compression ratio (CR) and the open porosity (*ϕ*) of the felt [Eq. (8)].

(7) $$d_{el}=\ln\left(\frac{I_{0}}{I}\right){\cdot \mu }^{-1}\cdot \phi_{r}$$

(8) $$\phi_{r}=\frac{CR-\phi }{CR-1}$$

Two main corrections were applied to the radiography. The first one is the beam intensity decay correction, applied after the example of Hinebaugh et al.^48^ Because the intensity of the X‐ray beam at the synchrotron decays over time, the background (flat field image) changes over time as well. As they described, taking only the background before the scan into account will lead to an overestimation of the saturation because the image appears as if more intensity was attenuated. Thus, the flat fields taken after the radiography scan were divided by the flat fields recorded before the scan (in both cases, darkfield subtraction before division), which resulted in a decay factor. Owing to the approximated linearity of the decay over a short period (scans are not longer than 7.5 min), the factor could be calculated for any given time. This time‐resolved factor was then applied to the radiogram at the corresponding time.

The second correction is the flat field and dark field correction. Darkfield images (DF) were subtracted from each radiogram to account for the camera noise. To apply the Beer–Lambert law to determine the electrolyte thickness, images of the dry cell (before injection with electrolyte, IMG_dry cell_) were recorded as the background. The grayscale values of each pixel of the radiogram (IMG_wet cell_) were subsequently divided by the grayscale values of the corresponding pixel in the background image, which consequently resulted in images containing solely the electrolyte because the division rids the image of any flat field effects of the cell setup. Equation (9) summarizes the processing:

(9) $$\frac{I}{I_{0}}=\frac{IMG_{wet\_cell\;}-DF}{IMG_{dry\_cell}-DF}$$

These radiograms were then ready to enter the Beer–Lambert law as the *I* over *I*_0_ term. Local saturation and average saturation values were calculated by using a python script written in‐house.

After the data analysis was completed, thresholds were applied to omit noise in the 3D images and the contrast was enhanced to discern the multiple phases by using the open‐source imaging tool package Fiji® (based on ImageJ).

To additionally increase the visibility of the saturation values of the averaged radiograph frames, recorded during cyclic voltammetry and hydrogen evolution experiments, LOWESS was applied with a data range of 0.03 and 0.01, respectively.

The tomograms were visualized in Avizo® by the means of the functions Median Filter and Interactive Thresholding.

## Conflict of interest

*The authors declare no conflict of interest*.
